# Supplementary material for: Linking Self-Control to Negative Risk-Taking Behavior among Chinese Late Adolescents: A Moderated Mediation Model
Source: Int J Environ Res Public Health. 2022 Jun 22;19(13):7646. doi: 10.3390/ijerph19137646 (PMC9265433; doi:10.3390/ijerph19137646)
Supplement: Supplementary file 1 [file ijerph-19-07646-s001.zip › ijerph-1711507-supplementary.pdf]

Supplementary Materials

Adolescent Risk-taking Questionnaire

Below are seventeen statements with which you may do always or never. Using the 5-scale below, indicate your participation in the bellowing activities by placing the appropriate number on the line preceding that item. Please be open and honest in your responding. The 5-point scale is as follows:

0 = never do
1 = hardly ever do
2 = do sometimes
3 = do often
4 = do very often

____ 1. Leaving school
____ 2. Underage drinking
____ 3. Smoking
____ 4. Getting drunk
____ 5. Take Drugs
____ 6. Staying out late
____ 7. Drinking and driving
____ 8. Have unprotected sex
____ 9. Overeating
____ 10. Teasing and picking on people
____ 11. Cheating

Brief Self-Control Scale

Using the scale provided, please indicate how much each of the following statements reflects how you typically are.

1 = not like me at all
2 = slightly like me
3 = moderately like me
4 = fairly like me
5= very much like me

____ 1. I am good at resisting temptation.
____ 2. I have a hard time breaking bad habits. (r)
____ 3. I am lazy. (r)
____ 4. I say inappropriate things. (r)
____ 5. I do certain things that are bad for me, if they are fun. (r)
____ 6. I refuse things that are bad for me.
____ 7. I wish I had more self-discipline. (r)
____ 8. People would say that I have iron self- discipline.
____ 9. Pleasure and fun sometimes keep me from getting work done. (r)
____ 10. I have trouble concentrating. (r)
____ 11. I am able to work effectively toward long-term goals.
____ 12. Sometimes I can’t stop myself from doing something, even if I know it is wrong.
____ 13. I often act without thinking through all the alternatives. (r)

Regulatory Focus Questionnaire

This set of questions asks you about specific events in your life. Please indicate your answer to each question by circling the appropriate number below it.

1 = never or seldom
2 = rarely
3 = sometimes
4 = often
5= very often

____ 1. Compared to most people, are you typically unable to get what you want out of life?
____ 2. Growing up, would you ever ``cross the line" by doing things that your parents would not tolerate?
____ 3. How often have you accomplished things that got you ``psyched" to work even harder?
____ 4. Did you get on your parents' nerves often when you were growing up?
____ 5. How often did you obey rules and regulations that were established by your parents?
____ 6. Growing up, did you ever act in ways that your parents thought were objectionable?
____ 7. Do you often do well at different things that you try?
____ 8. Not being careful enough has gotten me into trouble at times.
____ 9. When it comes to achieving things that are important to me, I find that I don't perform as well as I ideally would like to do.
____ 10. I feel like I have made progress toward being successful in my life.
____ 11. I have found very few hobbies or activities in my life that capture my interest or motivate me to put effort into them.

Personal Sense of Power Scale

In rating each of the items below, please use the following scale:

1 = disagree strongly
2 = disagree
3 = disagree a little
4 = neither agree nor disagree
5 = agree a little
6 = agree
7 = agree strongly

____ 1. I can get him/her/them to listen to what I say.
____ 2. My wishes do not carry much weight. (r)
____ 3. I can get him/her/them to do what I want.
____ 4. Even if I voice them, my views have little sway. (r)
____ 5. I think I have a great deal of power.
____ 6. My ideas and opinions are often ignored. (r)
____ 7. Even when I try, I am not able to get my way. (r)
____ 8. If I want to, I get to make the decisions.
